# Cerebrospinal fluid and plasma biomarkers in idiopathic normal pressure hydrocephalus: comparison with Alzheimer's and Parkinson's diseases

**DOI:** 10.3389/fnagi.2026.1866009

**Published:** 2026-08-24

**Authors:** Francesca Pappafava, Lorenzo Gaetani, Matteo Maria Ottaviani, Giulia Fiorucci, Giovanna Nardi, Nicola Salvadori, Alfredo Megaro, Federico Paolini Paoletti, Lucilla Parnetti, Carlo Conti

**Affiliations:** 1 University of Perugia, Perugia, Italy; 2 Section of Neurology, Department of Medicine and Surgery, University of Perugia, Perugia, Italy; 3 Neurosurgery Unit, Struttura Complessa Interaziendale di Neurochirurgia Perugia-Terni, Azienda Ospedaliera di Perugia, Perugia, Italy

**Keywords:** Alzheimer's disease, biomarker, cerebrospinal fluid, idiopathic normal pressure hydrocephalus, Parkinson's disease, plasma, Hakim disease

## Abstract

**Background:**

Idiopathic normal pressure hydrocephalus (iNPH) is a potentially reversible neurological disorder whose diagnosis is complicated by clinical overlap with neurodegenerative diseases. Fluid biomarkers may provide insight into disease mechanisms and support differential diagnosis.

**Methods:**

We performed a small exploratory study at the Neurology and Neurosurgery Sections of the University Hospital of Perugia, between 2024 and 2025, comparing cerebrospinal fluid (CSF) and plasma biomarkers among patients with iNPH, Alzheimer's disease (AD), Parkinson's disease (PD), and non-neurodegenerative controls (CTRL). CSF concentrations of amyloid-β peptides (Aβ42, Aβ40, Aβ42/Aβ40 ratio), tau proteins (p-tau 181 and t-tau) and neurofilament light chain (NfL) were measured. In plasma, amyloid-β peptides (Aβ42, Aβ40, Aβ42/Aβ40 ratio), tau proteins (p-tau 217), neurofilament light chain (NfL), and glial fibrillary acidic protein (GFAP) were assessed. Both CSF and plasma biomarkers were measured using standardized immunoassays. Group differences and correlations between CSF and plasma biomarkers were analyzed.

**Results:**

CSF Aβ40 levels were significantly lower in iNPH compared with all other groups (all p < 0.005), and showed preliminary apparent discrimination for iNPH, requiring external validation, with an AUC of 0.95, sensitivity of 95%, and specificity of 86% at a cut-off of 9,327.5 pg/mL. AD patients were clearly distinguished from iNPH and the other groups by AD-specific changes in fluid biomarkers. CSF Aβ42 levels were lower in iNPH than in CTRL (*p* = 0.001) and PD (*p* = 0.009), while CSF p-tau181 and t-tau were decreased compared with AD (*p* < 0.001) and CTRL (*p* ≤ 0.024). The CSF Aβ42/Aβ40 ratio remained preserved in iNPH, distinguishing it from AD (*p* < 0.001).

Plasma NfL correlated strongly with CSF levels in each diagnostic group, including iNPH (*r* = 0.77, *p* = 0.0012), whereas plasma amyloid biomarkers poorly reflected CSF concentrations.

**Conclusions:**

iNPH is associated with low CSF Aβ40 levels, possibly reflecting altered CSF dynamics rather than a primary pathophysiological mechanism. In iNPH, plasma NfL showed a preliminary correlation with CSF NfL; however, larger age-adjusted studies are required before peripheral markers can be considered clinically informative in this setting.

## Introduction

Idiopathic normal pressure hydrocephalus (iNPH) is a potentially reversible neurological disorder, that mainly affects people over 65 years old. It was first described in 1965 by Hakim and Adams (Hakim and Adams, 1965) as a syndrome characterized by ventriculomegaly and a clinical triad of gait disturbance, cognitive impairment, and urinary incontinence, known as Hakim-Adams Triad (Campo-Caballero et al., 2025).

Despite its treatable nature, with a shunt surgery (Nakajima et al., 2021), iNPH remains underdiagnosed and frequently misclassified, largely due to its clinical overlap with common neurodegenerative diseases such as Alzheimer's disease (AD) and Parkinson's disease (PD), and, in some cases, also due to the coexistence of iNPH with other diseases (Müller-Schmitz et al., 2020; Cabral et al., 2011; Shimada et al., 2025; Giliberto et al., 2017). iNPH is considered a disorder of cerebrospinal fluid (CSF) hydrodynamics, in which alterations in CSF production (Orešković et al., 2017; Miyajima and Arai, 2015), absorption (Miyajima and Arai, 2015; Bulat et al., 2008), and circulation (Luetmer et al., 2002; Yin et al., 2017; Broggi et al., 2025), dysfunction of the ependymal cilia (Ji et al., 2022; Banizs et al., 2005), and dysfunction of the blood-CSF and brain-CSF barriers have all been proposed as contributors (Mani et al., 2024; Wang et al., 2020). In particular, impaired glymphatic clearance (Reeves et al., 2020; Bae et al., 2021) and disrupted solute transport have been hypothesized to influence CSF composition and biomarker distribution, potentially distinguishing iNPH from other neurodegenerative diseases.

CSF biomarkers have become central tools for characterizing neurodegenerative diseases. Reduced CSF amyloid-beta 42 (Aβ42) concentration and Aβ42/amyloid-beta 40 (Aβ40) ratio, together with increased phosphorylated tau at threonine 181 (p-tau181) and total tau (t-tau) levels, are well-established hallmarks of AD, while increased CSF neurofilament light chain (NfL) and glial fibrillary acidic protein (GFAP) reflect axonal injury and astroglial activation across a range of neurological disorders (Blennow et al., 2016; Wojdała et al., 2023). Several of these markers can also be measured in blood, enabling less invasive biological characterization through simple blood sampling (Palmqvist et al., 2025). Recent reviews highlight that fluid biomarkers across CSF, blood, saliva, urine, and extracellular vesicles may improve early diagnosis and monitoring of neurodegenerative diseases, while still requiring standardization and multimodal validation (Cao et al., 2025).

The CSF biomarker profile of iNPH remains incompletely defined, and its distinction from overlapping neurodegenerative conditions continues to be debated. Moreover, the extent to which plasma biomarkers reflect central nervous system pathology in iNPH is still unclear, particularly in the context of altered CSF-blood barrier function. A narrative review of iNPH CSF biomarkers emphasized that no definitive iNPH CSF profile has been established, although low Aβ42 with relatively low tau compared with AD is frequently reported (Pyrgelis et al., 2022).

The aim of this exploratory pilot study was to characterize the CSF and plasma biomarker profile of patients with iNPH and to compare it with that of patients with AD, PD, and non-neurodegenerative controls (CTRL). Secondarily, we explored correlations between CSF and plasma biomarkers in the entire cohort and in the iNPH subgroup. Given the limited sample size and observational design, all analyses were considered hypothesis-generating and were not intended to support definitive clinical decision-making.

## Methods

### Study design and participants

This exploratory observational study was conducted at the Neurology and Neurosurgery Sections of the University Hospital of Perugia. The study included prospectively enrolled iNPH patients (*n* = 14), who underwent CSF collection as part of their diagnostic or therapeutic evaluation between 2024 and 2025. Retrospective comparison groups of patients diagnosed with AD (*n* = 10), PD (*n* = 10), and CTRL (*n* = 10) were also included. The diagnosis of iNPH was established according to current Japanese guidelines (Nakajima et al., 2021), based on clinical presentation, neuroimaging findings, and CSF tap-test response when available. However, complete data on the number of tap-test responders, the number of patients who underwent shunt surgery, and the number of shunt responders were not available in the present dataset; consequently, biomarker associations with tap-test response or shunt outcome could not be analyzed. AD and PD diagnoses were made according to established clinical diagnostic criteria (Jack et al., 2018; Postuma et al., 2015). Control subjects were individuals undergoing lumbar puncture for diagnostic purposes in whom neurological disease was excluded. The control group included individuals presenting with minor neurological conditions (i.e., mononeuropathy, headache, subjective cognitive complaints), who underwent lumbar puncture as part of their routine diagnostic work-up. Only subjects with a CSF profile not consistent with AD pathology and who did not meet criteria for any neurodegenerative disease were included. No patient demonstrated evidence of renal dysfunction.

Exclusion criteria for all groups included evidence of secondary causes of hydrocephalus, active inflammatory or infectious neurological disease, history of major cerebrovascular events, or conditions known to significantly affect CSF biomarker levels.

All patients underwent a standardized neurological evaluation. In the iNPH group, clinical severity was assessed using iNPH grading scale for gait, cognition, and functional status, including the Mini-Mental State Examination (MMSE), HIV-Dementia Scale (HDS), and Tinetti gait and balance scale, when available. Neuroimaging parameters such as Evan's index were recorded as part of routine diagnostic workup.

The study was conducted in accordance with the Declaration of Helsinki and approved by the local ethics committee. Written informed consent was obtained from all participants or their legal representatives.

Demographic and clinical characteristics of the study population are summarized in Table 1. AD, PD, and CTRL patients were retrospectively selected from the local clinical database with the aim of approximating the age and sex distribution of the prospectively enrolled iNPH cohort. Diagnostic groups did not differ significantly in age (*p* = 0.94) or sex distribution (*p* = 0.452); however, the absence of a statistically significant difference in a small sample does not exclude residual confounding, and unadjusted comparisons should be interpreted with this caveat in mind.

**Table 1:** Demographic and clinical characteristics of the study population.

Diagnosis	Subcategory	iNPH *n* = 14	AD *n* = 10	PD *n* = 10	CTRL *n* = 10
Age		78 (68–82)	74.5 (72–76.5)	73 (71.2–75.8)	73.5 (72.2–76.5)
Sex (%)	M	10 (71.4)	5 (50.0)	5 (50.0)	4 (40.0)
	F	4 (28.6)	5 (50.0)	5 (50.0)	6 (60.0)
ATN (%)	A-T-	11 (78.6)	0 (0.00)	5 (50.0)	5 (50.0)
	A-T+	1 (7.1)	0 (0.00)	3 (30.0)	4 (40.0)
	A+T-	1 (7.1)	0 (0.00)	1 (10.0)	1 (10.0)
	A+T+	1 (7.1)	10 (100.0)	1 (10.0)	0 (0.00)
MMSE_pre-TT		23 (19–25)			
MMSE			25 (20.2–26.8)	27.5 (24.2–28)	25.5 (22.2–27.8)
MMSE_post-TT		26 (23–29)			
Tinetti_pre-TT		18.50 ± 7.7			
Tinetti_post-TT		26 (18.5–28)			
HDS_pre-TT		8.06 ± 4.7			
HDS_post-TT		8.94 ± 3.6			
Evan's Index		0.37 ± 0.1			
UPDRS				25 (24.5–32.5)	
H and Y				2.21 ± 0.5	

Data are presented as mean ± SD or median (IQR) according to data distribution.

### CSF and plasma collection and analysis

Lumbar puncture was performed according to international guidelines. (Teunissen et al., 2009) 10 to 12 mL of CSF were collected in sterile polypropylene tubes (Sarstedt, cat#62.610.210), centrifuged for 10 min at 2000 x *g*, at room temperature, aliquoted into 0.5 mL polypropylene tubes (Sarstedt, cat#72.730.007) and stored at −80 °C. Before use, CSF samples were shortly thawed at room temperature (15 °C to 25 °C) for 30 min and vortexed for 10 s. Aβ42, Aβ40, t-tau, and p-tau181 measurements were performed in all the samples using fully automated CLEIA on the LUMIPULSE^®^ G System (LUMIPULSE^®^ G1200), according to manufacturer instructions at the Laboratory of Clinical Neurochemistry, Section of Neurology, University of Perugia. All CSF samples were analyzed directly in their 0.5 mL storage tubes. To determine A+ and T+ statuses, internally generated cut-offs were used (Bellomo et al., 2021). Blood samples were collected at the time of lumbar puncture, following international guidelines and standard operating procedures (Teunissen et al., 2009). Briefly, they were collected in BD VacutainerTM anti-coagulated ethylene-diamine-tetra-acetic acid (EDTA), centrifuged for 10 min at 2000 x *g*, aliquoted into 0.5 mL polypropylene tubes (Sarstedt, cat#72.730.007) and stored at −80 °C. Before use, plasma samples were shortly thawed at room temperature (15 °C to 25 °C) for 30 min, vortexed for 10 s, and centrifuged for 5 min at 2000 x *g*. Aβ42, Aβ40, phosphorylated tau at threonine 217 (p-tau217), NfL and GFAP measurements were performed using fully automated CLEIA on the LUMIPULSE^®^ G System (LUMIPULSE^®^ G1200), according to manufacturer instructions at the Laboratory of Clinical Neurochemistry, Section of Neurology, University of Perugia. All plasma samples were analyzed directly in their 0.5 mL storage tubes.

All CSF and plasma analyses were performed in the same laboratory on the same LUMIPULSE G platform and according to the same local standard operating procedures. All samples, including those from the retrospective groups, underwent a single freeze-thaw cycle and the same standardized thawing and processing procedure immediately before biomarker determination. Sampling storage duration however was not fully matched across groups, and formal batch-effect analyses were not performed. This potential preanalytical heterogeneity was considered in the interpretation of the results.

### Statistical analysis

All data were entered into a dedicated database and analyzed using RStudio (version: 2025.09.1+401; Copyright (C) 2025 by Posit Software, PBC). Descriptive statistics were used to summarize demographic, clinical, and biochemical variables. Continuous variables are reported as mean ± standard deviation (SD), and categorical variables are presented as frequencies and percentages. Biomarker distributions were assessed for normality, and log10 transformation was applied when required, in particular for CSF biomarkers: Aβ42, p-tau181 and t-tau and plasma biomarkers: NfL and GFAP. Group comparisons were performed using one-way analysis of variance (ANOVA) with *post hoc* Tukey correction or the Kruskal-Wallis test with Dunn's multiple comparisons, depending on data distribution. For Dunn-test comparisons, effect estimates and unadjusted 95% confidence intervals were derived using the Hodges-Lehmann estimator from the corresponding pairwise Wilcoxon rank-sum test. Because the Dunn-test *p*-values were Bonferroni-adjusted whereas these confidence intervals were not multiplicity-adjusted, an interval excluding the null may occasionally accompany a non-significant adjusted *p*-value; statistical significance was determined from the adjusted *p*-value. Effect sizes were calculated for all pairwise comparisons (Cohen's d for ANOVA-based comparisons; rank-biserial correlation *r* for Kruskal-Wallis/Dunn-based comparisons). Overall *p*-values were further corrected for multiple comparisons across biomarkers within each panel (CSF, plasma) using the Benjamini-Hochberg false discovery rate (FDR) procedure. Correlations between CSF and plasma biomarker levels were assessed using Pearson or Spearman correlation coefficients, as appropriate. Given the small number of paired observations in the iNPH group, CSF-plasma NfL correlations were interpreted as exploratory; formal age-adjusted and outlier-removal sensitivity analyses were not considered sufficiently powered. Receiver operating characteristic (ROC) curve analysis was performed as an exploratory assessment of the diagnostic performance of CSF Aβ40 in distinguishing iNPH from AD and PD considered together, reflecting the clinically relevant differential diagnosis scenario between a potentially shunt-responsive condition and irreversible neurodegenerative disease. Healthy controls were not included in this comparison. To assess the stability of the selected diagnostic cut-off and the corresponding out-of-sample sensitivity, specificity, and accuracy, we performed a leave-one-out cross-validation (LOOCV) analysis. The ROC AUC was reported only as an apparent, full-sample estimate and was not considered independently cross-validated by this procedure. For each iteration, one patient was excluded from the dataset, and the optimal cut-off value (Youden index) was re-calculated from the receiver operating characteristic (ROC) curve generated on the remaining *n*-1 patients. The excluded patient was then classified according to this newly derived threshold. This procedure was repeated for all patients, yielding an out-of-sample sensitivity, specificity, and accuracy, as well as a distribution of threshold values across iterations. A *p*-value < 0.05 was considered statistically significant.

## Results

### CSF biomarker profiles

**Figure 1:**
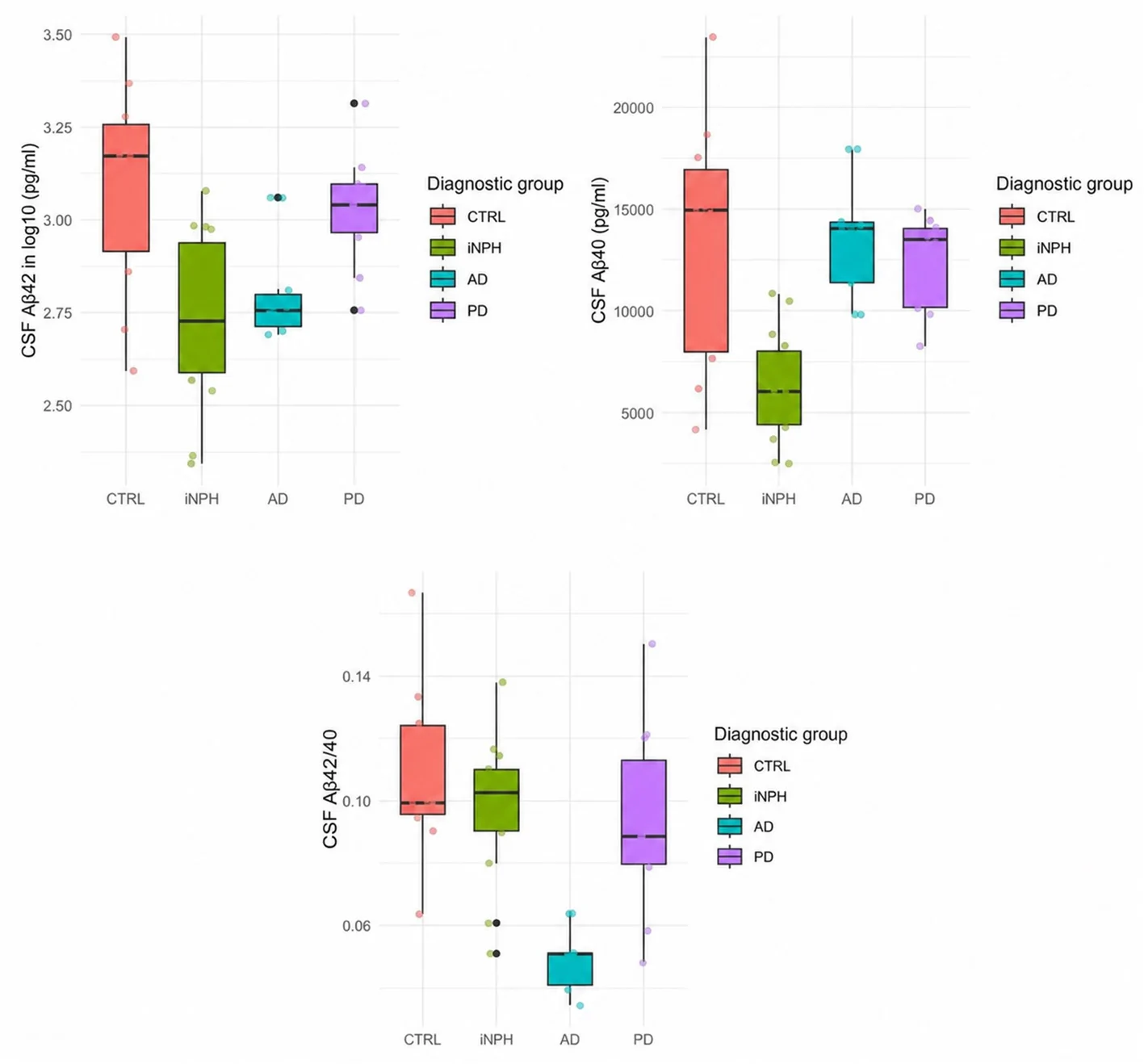
Comparison of CSF Aβ42 and Aβ40 concentrations (pg/mL) and the dimensionless CSF Aβ42/Aβ40 ratio among groups.

Significant group differences were observed for CSF Aβ42, Aβ40, Aβ42/Aβ40 ratio, p-tau181, and t-tau (Table 2), whereas CSF NfL did not differ significantly between groups, with an FDR adjusted *p*-value of 0.53 in the ANOVA test. Log10-transformed CSF Aβ42 levels were significantly lower in iNPH compared with CTRL (*p* = 0.001) and PD (*p* = 0.009), and in AD compared with CTRL (*p* = 0.023). No significant differences were observed between AD and PD, iNPH and AD or between PD and CTRL (Figure 1).

**Table 2:** CSF biomarker concentrations across diagnostic groups.

CSF biomarkers	Group^1^(pg/mL)	Comparison	Difference	Confidence interval	Effect-size	*p*-value
Aβ42 (pg/mL) *p*-value: < 0.001 (FDR adjusted *p*-value: < 0.001)	**iNPH:** 534 (389–876) **AD:** 570 (516–631) **PD:** 1097 (924–1247) **CTRL:** 1485 (842–1811)	iNPH–AD	−0.072	(−0.31; 0.16)	−0.383	0.84 ^#^
		iNPH–PD	−0.30	(−0.53; −0.06)	−1.51	0.009 ^#^
		iNPH–CTRL	−0.36	(−0.6; −0.12)	−1.38	0.001 ^#^
		PD–AD	0.22	(−0.03; 0.48)	+1.51	0.11 ^#^
		PD–CTRL	−0.06	(−0.32; 0.19)	−0.276	0.9 ^#^
		CTRL–AD	0.27	(0.03; 0.54)	1.27	0.023 ^#^
Aβ40 (pg/mL) *p*-value: < 0.001 (FDR adjusted *p*-value: < 0.001)	**iNPH:** 6252 ± 2685 **AD:** 13513 ± 2932 **PD:** 12293 ± 2402 **CTRL:** 13165 ± 6182	iNPH–AD	−7260.8	(−11,439.1; −3082.5)	−2.58	0.0002 ^#^
		iNPH–PD	−6040.8	(−10,219.1; −1862.5)	−2.37	0.002 ^#^
		iNPH–CTRL	−6913.13	(−11,091.43; −2,734.8)	−1.45	0.0004 ^#^
		PD–AD	−1220	(−5,733.1; 3,293.1)	−0.455	0.89 ^#^
		PD–CTRL	−872.33	(−5,385.4; 3,641)	−0.186	0.95 ^#^
		CTRL–AD	−347.67	(−4,861; 4,165.4)	−0.0719	0.99 ^#^
Aβ42/Aβ40 *p*-value: < 0.001 (FDR adjusted *p*-value: < 0.001)		iNPH–AD	0.05	(0.02; 0.08)	2.8	0.0001 ^#^
		iNPH–PD	0.005	(−0.02; 0.03)	0.18	0.96 ^#^
		iNPH–CTRL	−0.012	(−0.04; 0.02)	−0.45	0.65 ^#^
		PD–AD	0.043	(0.015; 0.07)	+1.94	0.001 ^#^
		PD–CTRL	−0.016	(−0.05; 0.01)	−0.556	0.43 ^#^
		CTRL–AD	0.06	(0.03; 0.09)	2.83	< 0.001 ^#^
p-tau181 (pg/mL) *p*-value: < 0.001 (FDR adjusted *p*-value: < 0.001)	**iNPH:** 23.6 (21.4–37.9) **AD:** 101.8 (76–125.7) **PD:** 43.8 (34.2–64.2) **CTRL:** 49 (43.4–59)	iNPH–AD	−0.55	(−0.78; −0.34)	−2.56	< 0.001^#^
		iNPH–PD	−0.2	(−0.42; 0.01)	−0.987	0.07 ^#^
		iNPH–CTRL	−0.24	(−0.46; −0.02)	−1.17	0.024 ^#^
		PD–AD	−0.35	(−0.6; −0.12)	−2.12	0.0012 ^#^
		PD–CTRL	−0.036	(−0.3; 0.2)	−0.243	0.97 ^#^
		CTRL–AD	−0.32	(−0.55; −0.09)	−1.9	0.004 ^#^
t-tau (pg/mL) *p*-value: < 0.001 (FDR adjusted *p*-value: < 0.001)	**iNPH:** 218 (191–399) **AD:** 675 (530–1057) **PD:** 309 (267–449) **CTRL:** 329 (275–424)	iNPH–AD	−0.44	(−0.7; −0.19)	−1.89	0.0002 ^#^
		iNPH–PD	−0.12	(−0.37; 0.14)	−0.449	0.61 ^#^
		iNPH–CTRL	−0.094	(−0.35; 0.16)	−0.425	0.75 ^#^
		PD–AD	−0.33	(−0.6; −0.05)	−1.44	0.014 ^#^
		PD–CTRL	0.02	(−0.25; 0.3)	0.0992	0.99 ^#^
		CTRL–AD	−0.35	(−0.62; −0.07)	−1.88	0.008 ^#^
NfL (pg/mL) (FDR adjusted *p*-value: 0.53)	**iNPH:** 1115 ± 787 **AD:** 950 ± 248	iNPH–AD	165.3	(−371.63; 702.23)	0.283	0.53 (ANOVA)

Data are presented as mean ± SD or median (interquartile range)^1^, according to data distribution. Group differences were assessed using one-way ANOVA with Tukey *post hoc* correction (#) or the Kruskal-Wallis test with Dunn *post hoc* correction (Bonferroni-adjusted) (§), as appropriate. Omnibus *p*-values were further adjusted for multiple comparisons across biomarkers using the Benjamini-Hochberg false discovery rate (FDR). Effect sizes (Cohen's *d* for ANOVA-based comparisons; *r* for Kruskal-Wallis-based comparisons) are reported alongside *p*-values. Significance was set at *p* < 0.05. For Kruskal-Wallis/Dunn comparisons, the reported Hodges-Lehmann 95% confidence intervals are unadjusted, whereas the displayed *p*-values are Bonferroni-adjusted; therefore, exclusion of the null by an interval may not correspond to statistical significance after multiplicity correction.

CSF Aβ40 levels were significantly lower in iNPH compared with CTRL, AD, and PD (*p* < 0.001, *p* < 0.001, and *p* = 0.002, respectively) (Table 2 and Figure 1).

An exploratory ROC curve analysis was performed to estimate the apparent diagnostic discrimination of CSF Aβ40 in distinguishing iNPH from the AD and PD groups considered together. CSF Aβ40 showed an apparent area under the curve (AUC) of 0.95 (95% CI: 0.883–1.000, DeLong method), a sensitivity of 95% (95% CI: 70.0–100%), and a specificity of 86% (95% CI: 71.4–100%), based on bootstrap resampling (2,000 replicates).

**Figure 2:**
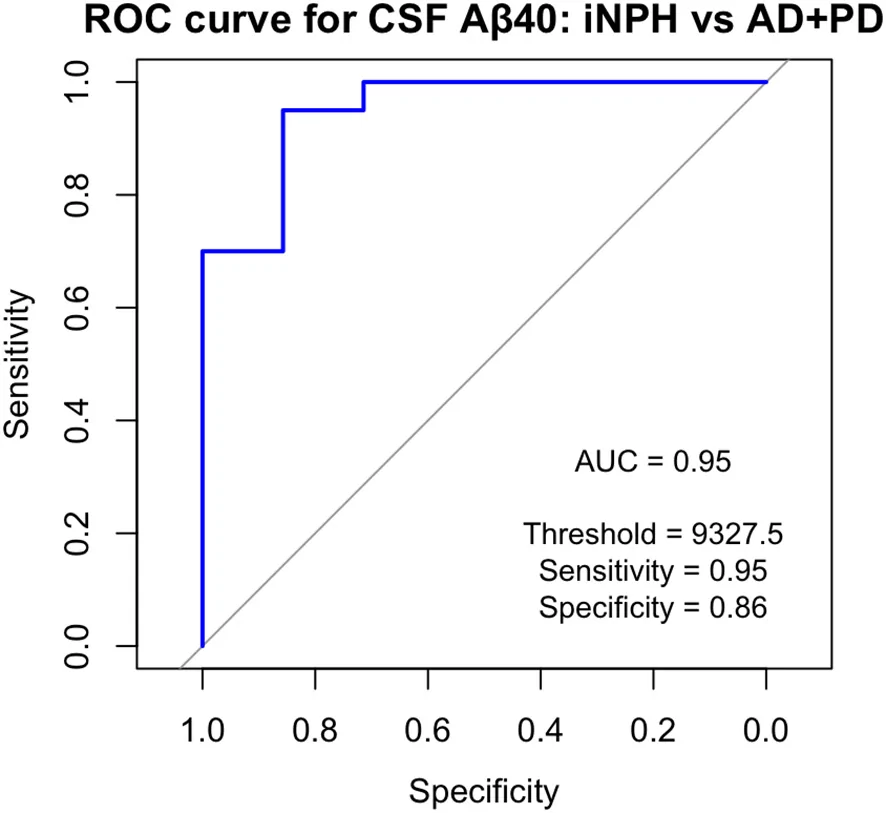
Apparent receiver operating characteristic (ROC) curve for CSF Aβ40 in distinguishing iNPH from AD and PD combined (comparator group).

A cut-off value of 9,327.5 pg/mL (95% CI: 7,635–11,130 pg/mL) optimally differentiated iNPH from AD and PD considered together in this cohort (Figure 2). Given the modest sample size, these confidence intervals are relatively wide and should be interpreted with caution; this cut-off should be regarded as preliminary and requires validation in larger, prospectively characterized cohorts.

LOOCV was used to evaluate the stability of the selected threshold and the corresponding out-of-sample classification performance. The LOOCV-derived sensitivity and specificity were 95.0% and 86%, respectively, with an overall accuracy of 91.2%; the re-estimated threshold remained stable across iterations (mean ± SD, 9,319 ± 49.1 pg/mL). The apparent AUC was 0.95. Because LOOCV reused the same observed biomarker values and was applied to threshold selection, it does not constitute independent validation of the AUC or external validation of the classifier.

The CSF Aβ42/Aβ40 ratio was higher in iNPH compared with AD and significantly lower in AD compared with CTRL and PD (all *p* ≤ 0.001), while no differences were observed among CTRL, iNPH, and PD (Figure 1).

**Figure 3:**
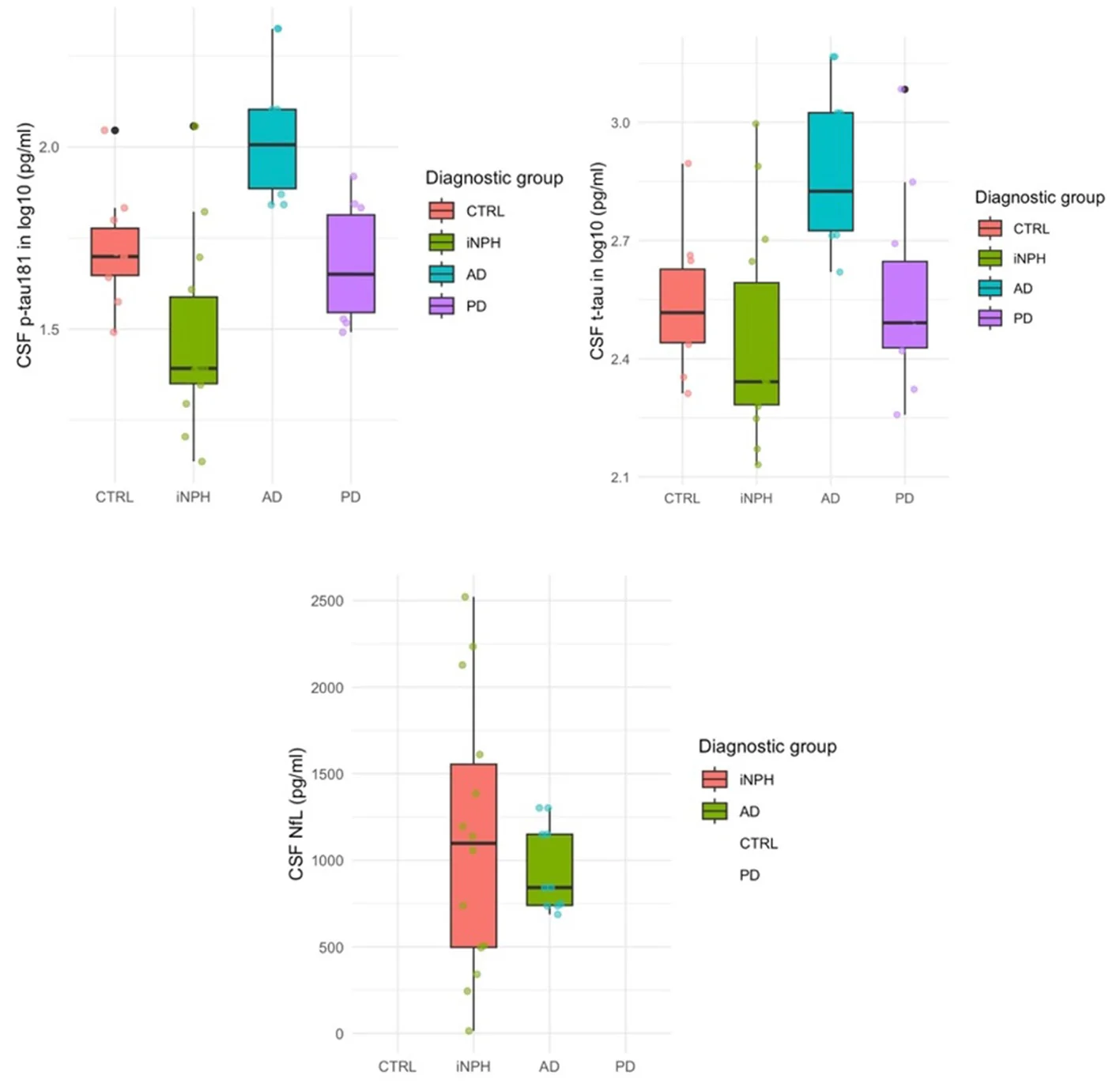
Comparison of CSF p-tau 181, t-tau and NfL levels among groups.

Log10-transformed CSF p-tau181 levels were lower in iNPH compared with AD and CTRL (*p* ≤ 0.01 and *p* = 0.024, respectively) with large effect sizes (Cohen's *d* = −2.56 and −1.17). Log10-transformed CSF t-tau levels were also lower in iNPH compared with AD (*p* < 0.001). No significant differences were found between PD and CTRL or between PD and iNPH for either biomarker (Figure 3). CSF NfL levels, analyzed in AD and iNPH only, did not differ significantly between groups (ANOVA *p* = 0.53) (Figure 3).

#### Plasma biomarker profiles

**Figure 4:**
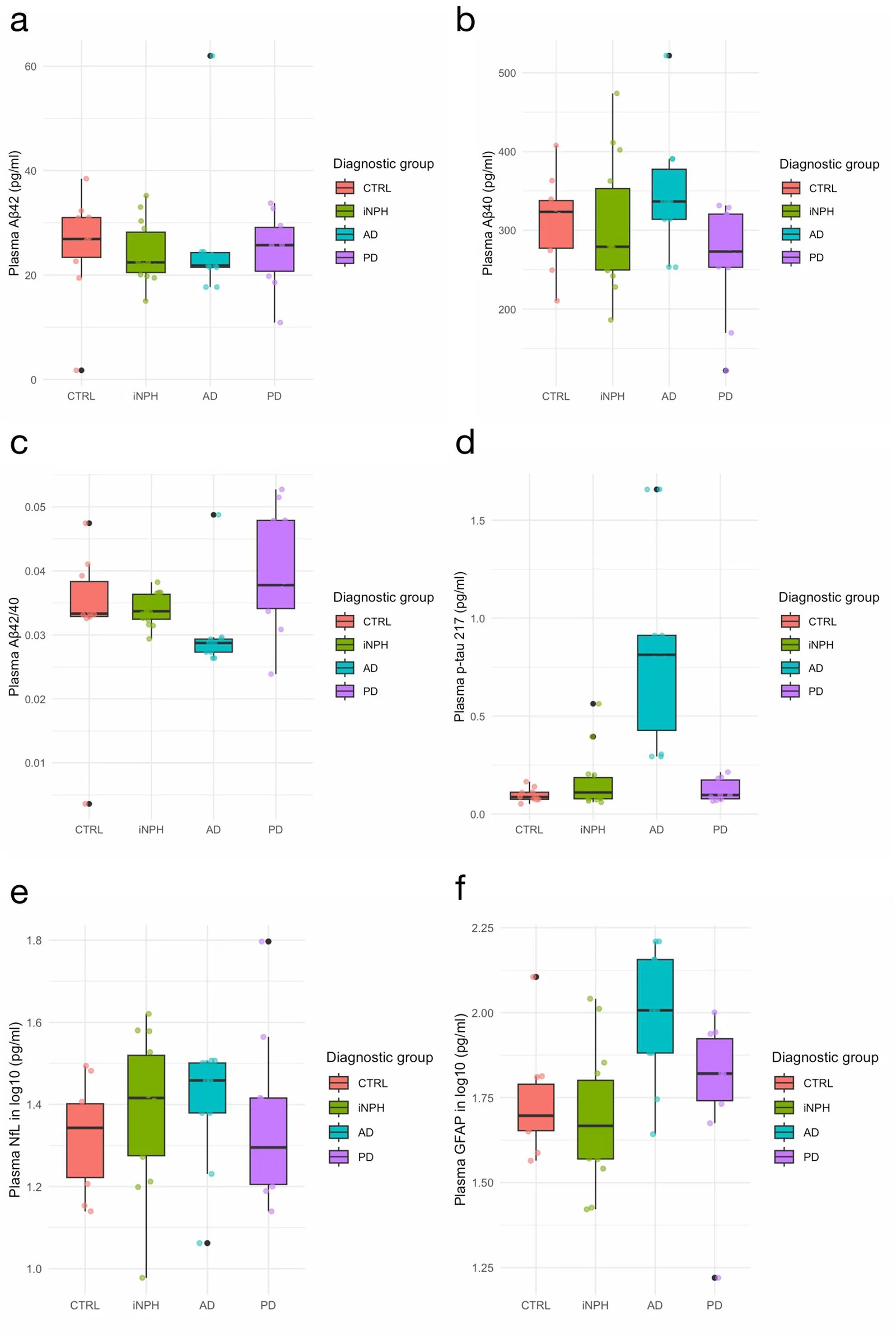
Comparison of plasma Aβ42 **(a)** and Aβ40 **(b)** concentrations (pg/mL), the dimensionless plasma Aβ42/Aβ40 ratio **(c)**, and plasma p-tau217 **(d)**, NfL **(e)**, and GFAP **(f)** concentrations (pg/mL) among groups.

Among plasma biomarkers, significant group differences were observed for GFAP, Aβ42/Aβ40 ratio, and p-tau217, whereas plasma NfL, Aβ42 and Aβ40 did not differ significantly across groups (ANOVA: *p* = 0.734, Kruskal: *p* = 0.44 and ANOVA: *p* = 0.125, respectively) (Figure 4) (Table 3).

**Table 3:** Plasma biomarker concentrations across diagnostic groups.

Plasma biomarkers	Group^1^(pg/mL)	Comparison	Difference	Confidence interval	Effect-size	p-value
Aβ42 (pg/mL) *p*-value: 0.44 (FDR adjusted *p*-value: 0.528)	**iNPH:** 22.4 (20.5–28.2) **AD:** 21.8 (21.5–24.3) **PD:** 25.7 (20.7–29.1) **CTRL:** 26.9 (23.4–31)	iNPH–AD	0.77	(−2.4; 5.9)	0.144	1^§^
		iNPH–PD	−1.14	(−6.37; 4.73)	−0.06	1^§^
		iNPH–CTRL	−3.21	(−8.52; 3.15)	−0.215	1^§^
		AD–PD	−2.43	(−8.13; 4.68)	−0.237	1^§^
		AD–CTRL	−4.82	(−9.47; 2.41)	−0.355	0.68^§^
		CTRL–PD	1.52	(−6.14; 8.53)	0.085	1^§^
Aβ40 (pg/mL) *p*-value: 0.125 (FDR adjusted *p*-value: 0.1875)	**iNPH:** 302 ± 82 **AD:** 345 ± 78 **PD:** 265 ± 70 **CTRL:** 311 ± 57	iNPH–AD	−42.56	(−123.93; 38.82)	−0.534	0.5^#^
		iNPH–PD	37.8	(−43.58; 119.17)	0.496	0.6^#^
		iNPH–CTRL	−8.7	(−90.02; 72.72)	−0.123	1^#^
		AD–PD	80.4	(−7.54; 168.24)	1.08	0.08^#^
		AD–CTRL	33.9	(−54; 121.8)	0.496	0.7^#^
		CTRL–PD	46.5	(−41.44; 134.34)	0.723	0.50^#^
Aβ42/Aβ40 *p*-value: 0.0047 (FDR adjusted *p*-value: 0.0094)		iNPH–AD	0.013	(0.008; 0.018)	0.658	0.076 ^§^
		iNPH–PD	−0.011	(−0.037; 0.001)	−0.359	1 ^§^
		iNPH–CTRL	−0.002	(−0.011; 0.007)	−0.072	1 ^§^
		AD–PD	−0.022	(−0.052, −0.009)	−0.559	0.003 ^§^
		AD–CTRL	−0.014	(−0.026; −0.009)	−0.525	0.098 ^§^
		CTRL–PD	−0.011	(−0.039; 0.007)	−0.304	1 ^§^
p-tau217 (pg/mL) *p*-value: < 0.001 (FDR adjusted *p-*value: < 0.001)	**iNPH:** 0.11 (0.08–0.19) **AD:** 0.81 (0.43–0.91) **PD:** 0.10 (0.08–0.17) **CTRL:** 0.09 (0.07–0.11)	iNPH–AD	−0.708	(−0.831; −0.228)	−0.766	0.001 ^§^
		iNPH–PD	0.002	(−0.039; 0.069)	0.018	1 ^§^
		iNPH–CTRL	0.015	(−0.015; 0.087)	0.185	1 ^§^
		AD–PD	0.713	(0.224; 0.842)	0.846	0.002^§^
		AD–CTRL	0.721	(0.221; 0.839)	0.846	0.0002 ^§^
		CTRL–PD	−0.015	(−0.078; 0.017)	−0.186	1 ^§^
NfL (pg/mL) *p*-value: 0.734 (FDR adjusted *p*-value: 0.734)	**iNPH:** 25.1 (17.8–32.1) **AD:** 27.7 (22.9–30.7) **PD:** 18.9 (15–25) **CTRL:** 21 (15.7–24.2)	iNPH–AD	−0.012	(−0.2; 0.17)	−0.0739	1^#^
		iNPH–PD	0.033	(−0.16; 0.22)	0.170	1^#^
		iNPH–CTRL	0.064	(−0.12; 0.25)	+0.406	0.8^#^
		AD–PD	0.045	(−0.16; 0.25)	0.254	0.93^#^
		AD–CTRL	0.08	(−0.13; 0.28)	+0.554	0.74^#^
		CTRL–PD	−0.03	(−0.23; 0.17)	−0.181	0.97^#^
GFAP (pg/mL) *p*-value: 0.0041 (FDR adjusted *p*-value: 0.0094)	**iNPH:** 45.75 (36.12–62.45) **AD:** 100.60 (75.10–142.30) **PD:** 65.20 (54.12–82.95) **CTRL:** 48.80 (43.98–60.80)	iNPH–AD	−0.30	(−0.52; −0.09)	−1.54	0.003 ^#^
		iNPH–PD	−0.092	(−0.31; 0.12)	−0.445	0.67 ^#^
		iNPH–CTRL	−0.04	(−0.26; 0.17)	−0.249	0.94 ^#^
		AD–PD	0.21	(−0.02; 0.44)	0.996	0.089 ^#^
		AD–CTRL	0.26	(0.03; 0.5)	1.45	0.023 ^#^
		CTRL–PD	−0.05	(−0.3; 0.18)	−0.255	0.94 ^#^

Data are presented as mean ± SD or median (interquartile range)^1^, according to data distribution. Group differences were assessed using one-way ANOVA with Tukey *post hoc* correction (#) or the Kruskal-Wallis test with Dunn *post hoc* correction (Bonferroni-adjusted) (§), as appropriate. Omnibus *p*-values were further adjusted for multiple comparisons across biomarkers using the Benjamini-Hochberg false discovery rate (FDR). Effect sizes (Cohen's *d* for ANOVA-based comparisons; *r* for Kruskal-Wallis-based comparisons) are reported alongside *p*-values. Significance was set at *p* < 0.05. For Kruskal-Wallis/Dunn comparisons, the reported Hodges-Lehmann 95% confidence intervals are unadjusted, whereas the displayed *p*-values are Bonferroni-adjusted; therefore, exclusion of the null by an interval may not correspond to statistical significance after multiplicity correction.

The log10-transformed plasma GFAP levels were significantly lower in iNPH compared with AD (*p* = 0.003), with no differences observed with PD and CTRL. In AD the level was higher compared with CTRL (*p* = 0.023), with no differences observed between PD and other groups. The overall difference across groups remained significant after FDR correction for multiple comparisons across the biomarker panel (FDR-adjusted *p* = 0.0094) (Figure 4). The plasma Aβ42/Aβ40 ratio showed no significant pairwise differences between iNPH and the other groups, and within the other groups, besides PD and AD, where the difference was significantly higher in PD compared with AD (*p* = 0.003) (Figure 4). Plasma p-tau217 levels were significantly lower in iNPH compared with AD (*p* = 0.0012) and elevated in AD compared with CTRL and PD (all *p* ≤ 0.002). No other differences were observed among CTRL, iNPH, and PD (Figure 4).

#### Correlations between CSF and plasma biomarkers

**Figure 5:**
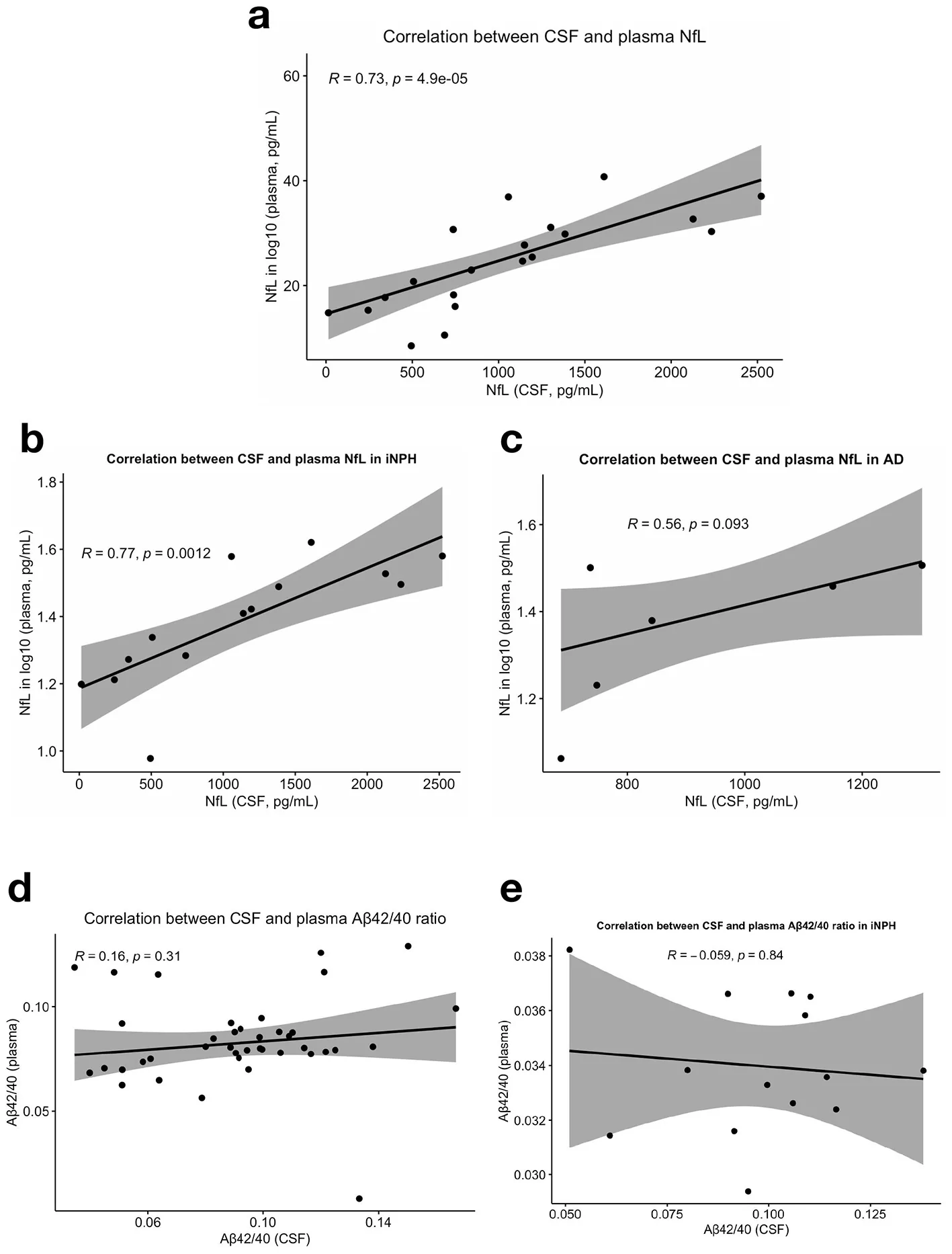
Correlations between CSF and plasma NfL concentrations in the combined iNPH and AD population **(a)**, the iNPH subgroup **(b)**, and the AD subgroup **(c)**, and between the dimensionless CSF and plasma Aβ42/Aβ40 ratios in the overall population **(d)** and the iNPH subgroup **(e)**. Variables displayed on a log10-transformed scale are identified explicitly in the corresponding axis labels.

For biomarkers measured in both CSF and plasma, correlation analyses were conducted to evaluate relationships between the two biofluids. A positive correlation was observed between plasma and CSF NfL levels in the combined iNPH and AD population (Pearson *r* = 0.73, *p* < 0.001) (Figure 5). In the iNPH subgroup, CSF and plasma NfL were also correlated (*r* = 0.77, *p* = 0.0012); however, this analysis included a small number of paired observations and may be influenced by age distribution or individual outliers. Therefore, this finding should be interpreted as exploratory and does not establish plasma NfL as a clinically reliable surrogate marker in iNPH. In contrast, no significant correlation was observed between CSF and plasma Aβ42/Aβ40 ratio in the overall population (*r* = 0.155, *p* = 0.315) (Figure 5). Subgroup analyses demonstrated a significant positive correlation only in PD (Spearman ρ = 0.70, *p* = 0.031), with no significant correlations in AD, iNPH, or CTRL.

## Discussion

This exploratory pilot study investigated CSF and plasma biomarkers across patients with iNPH, AD, PD, and CTRL, with the aim of describing candidate biomarker patterns rather than establishing clinically validated diagnostic criteria. The main finding was that CSF Aβ40 was significantly reduced in iNPH compared with all other groups, while the CSF Aβ42/Aβ40 ratio was preserved and tau levels were relatively low. This pattern argues against a typical AD-like CSF profile in most iNPH cases in this cohort, because AD is generally characterized by a reduced Aβ42/Aβ40 ratio accompanied by increased tau markers.

These findings should be interpreted in the context of previous iNPH biomarker literature. Prior studies have reported that iNPH can show low CSF Aβ42 despite absent amyloid PET pathology and that Aβ40 and Aβ42 may decrease proportionally, leaving the Aβ42/Aβ40 ratio relatively preserved (Kim et al., 2019). Other work has shown that iNPH is often characterized by low tau and low APP-derived proteins and that the combination of t-tau, Aβ40, and MCP-1 can separate iNPH from several clinical mimics (Jeppsson et al., 2019). Studies focusing on AD biomarkers in iNPH have further suggested that isolated low Aβ42 with a negative Aβ42/Aβ40 ratio and low tau may represent a typical iNPH profile, whereas an abnormal Aβ42/Aβ40 ratio, especially with elevated p-tau, should raise suspicion for coexisting AD pathology (Mazzeo et al., 2022). Our study extends this literature by examining a limited CSF and plasma candidate-biomarker panel in a cohort that included AD, PD, and CTRL comparison groups, and by identifying CSF Aβ40 reduction as the principal biomarker difference in iNPH in this dataset.

Although the exploratory ROC analysis suggested that CSF Aβ40 may help distinguish iNPH from the other diagnostic groups, this result should not be interpreted as a validated diagnostic threshold. The sample size was small, the iNPH cohort lacked systematic shunt-outcome confirmation, and preanalytical differences between prospectively and retrospectively collected groups cannot be excluded. Therefore, CSF Aβ40 should be considered a candidate adjunctive biomarker requiring external validation rather than a clinically actionable standalone test. This limitation is clinically relevant because AD CSF biomarkers, particularly the Aβ42/Aβ40 ratio and tau levels, have been reported to differ between tap-test responders and non-responders in iNPH (Pyrgelis et al., 2023).

From a pathophysiological perspective, reduced CSF Aβ40 in iNPH may reflect altered CSF dynamics, ventricular enlargement, or dilution/distribution effects, potentially related to impaired glymphatic clearance (Lidén et al., 2022; Bergström et al., 2024). However, this interpretation is plausible but not directly proven by the present data. Alternative explanations include altered amyloid precursor protein processing or production, impaired clearance from interstitial fluid to CSF, changes in blood-CSF or brain-CSF barrier function, ventricular volume-related effects on biomarker concentration, and coexisting neurodegenerative pathology. Future studies incorporating volumetric MRI, amyloid PET or neuropathological confirmation, standardized CSF dynamics measures, and shunt-response data will be needed to distinguish among these mechanisms. In a cohort of 53 iNPH patients, amyloid positivity and AD co-pathology were common, and higher t-tau/p-tau levels or an AD CSF profile were associated with more severe memory impairment (Pyrgelis et al., 2024).

Recent CSF proteomic studies further broaden the biological context of iNPH. A large-scale proteomic analysis found downregulation of synaptic and cell-adhesion proteins and upregulation of inflammatory and vimentin-related signals, suggesting ependymal and transependymal flow dysfunction (Kamalian et al., 2024). Most directly, Ying et al. used mass spectrometry-based CSF proteomics in iNPH and control cohorts, included 44 shunted patients, and showed that machine learning based on preoperative proteomic profiles could predict shunt outcome; QPCT and RBP4 were proposed as candidate biomarkers of shunt prognosis (Ying et al., 2026). Compared with such unbiased proteomic approaches, our study is narrower and lacks shunt-outcome validation, but it may help prioritize Aβ40 and the Aβ42/Aβ40 ratio for future outcome-anchored studies.

The CSF-plasma NfL correlation observed in the iNPH subgroup is biologically plausible but preliminary. NfL has been associated with symptom burden in iNPH and may reflect axonal injury, but prior outcome-focused work has reported weak or inconsistent associations with postoperative improvement (Saadaldeen et al., 2025). In the present cohort, the paired iNPH sample was small and not adequately powered for age-adjusted or outlier-removal sensitivity analyses. Thus, plasma NfL cannot be considered reliable for clinical use in iNPH based on this dataset alone.

Plasma biomarkers showed a more heterogeneous pattern. Plasma p-tau217 and GFAP were significantly elevated in AD compared with other groups, consistent with their roles as markers of amyloid-related tau pathology and astroglial activation, respectively. In contrast, plasma Aβ42, Aβ40, and NfL did not demonstrate significant group differences, and no significant correlation was observed between plasma and CSF Aβ42/Aβ40 ratio in the overall population. These findings support cautious interpretation of peripheral amyloid and neurodegeneration markers in iNPH until larger paired CSF-plasma studies are available. The choice of different tau biomarkers in CSF and plasma was based on the current evidence regarding their relative diagnostic performance in each biofluid. While p-tau181 is a well-established CSF biomarker for Alzheimer's disease, current evidence suggests that, in plasma, p-tau217 shows a higher diagnostic accuracy and enables earlier detection of AD pathology compared to p-tau181. Therefore, rather than using the same p-tau isoform across matrices, plasma p-tau217 was chosen based on its superior performance as a blood-based biomarker for AD.

## Limitations

Key limitations of this small exploratory pilot should be acknowledged. First, its mixed enrollment design should be noted: the iNPH cohort was prospective, while the AD, PD, and control cohorts were retrospective. Second, although diagnostic groups did not differ significantly in age or sex, the small sample size limits the ability to formally exclude residual confounding by these variables, particularly for age-sensitive biomarkers such as NfL; unadjusted comparisons should therefore be interpreted with appropriate caution. Third, the relatively wide confidence intervals around the sensitivity, specificity, and cut-off estimates for CSF Aβ40 reflect the modest sample size of this exploratory cohort and should be considered when interpreting its diagnostic performance. Fourth, despite standardized laboratory procedures, potential inconsistencies arising from batch effects, varying storage durations, collection timeframes, and incompletely verified historical freeze-thaw exposure in the retrospective groups cannot be entirely dismissed. Fifth, the diagnosis of iNPH was based on clinical and imaging features and tap-test response when available, but complete information on tap-test responders, shunted patients, and shunt responders was unavailable. This is a major limitation because iNPH is a treatable disorder and biomarker relationships with tap-test or shunt outcome would be highly clinically relevant. Sixth, formal age-adjusted and outlier-based sensitivity analyses for the CSF-plasma NfL correlation were not sufficiently powered, so this result should be considered preliminary. Seventh, structural differences between groups, particularly increased ventricular volume and altered CSF/brain tissue ratio in iNPH, may influence biomarker concentrations independently of disease-specific pathophysiology. Ventricular enlargement and altered CSF dynamics could introduce dilutional or distributional effects that confound direct comparisons with control and neurodegenerative cohorts. Finally, amyloid PET, neuropathology, and systematic markers of blood-CSF barrier integrity were not available, limiting our ability to distinguish iNPH-related CSF dynamics from coexisting neurodegenerative pathology or barrier dysfunction.

## Conclusion

In conclusion, in this exploratory pilot cohort, iNPH was associated with reduced CSF Aβ40, preserved CSF Aβ42/Aβ40 ratios, and relatively low tau levels. These findings are consistent with previous reports that iNPH can show globally reduced amyloid/APP-related CSF markers without an AD-like Aβ42/Aβ40 ratio or tau profile. CSF Aβ40 may be a candidate adjunctive marker for differentiating iNPH from selected neurodegenerative disorders, but the present data do not validate a clinical cut-off and should not be used as a standalone diagnostic tool.

Overall, the results generate the hypothesis that the iNPH biomarker pattern may be influenced by altered CSF dynamics, ventricular volume, clearance mechanisms, barrier function, and/or coexisting pathology. Larger prospective studies with standardized preanalytical handling, volumetric imaging, tap-test and shunt-outcome data, and broader proteomic validation are needed before clinical implementation.

## Data Availability

The original contributions presented in the study are included in the article/supplementary material, further inquiries can be directed to the corresponding author/s.
